# Contribution of working memory in multiplication fact network in children may shift from verbal to visuo-spatial: a longitudinal investigation

**DOI:** 10.3389/fpsyg.2015.01062

**Published:** 2015-07-23

**Authors:** Mojtaba Soltanlou, Silvia Pixner, Hans-Christoph Nuerk

**Affiliations:** ^1^Graduate Training Centre of Neuroscience/International Max Planck Research School for Cognitive and Systems NeuroscienceTuebingen, Germany; ^2^Knowledge Media Research CenterTuebingen, Germany; ^3^Department of Psychology, Eberhard Karls UniversityTuebingen, Germany; ^4^Institute of Applied Psychology, UMIT – The Health and Life Sciences UniversityHall in Tyrol, Austria; ^5^LEAD Graduate School, Eberhard Karls UniversityTuebingen, Germany

**Keywords:** multiplication, arithmetic, fact retrieval, operand errors, verbal working memory, visuo-spatial working memory

## Abstract

Number facts are commonly assumed to be verbally stored in an associative multiplication fact retrieval network. Prominent evidence for this assumption comes from so-called operand-related errors (e.g., 4 × 6 = 28). However, little is known about the development of this network in children and its relation to verbal and non-verbal memories. In a longitudinal design, we explored elementary school children from grades 3 and 4 in a multiplication verification task with the operand-related and -unrelated distractors. We examined the contribution of multiplicative fact retrieval by verbal and visuo-spatial short-term and working memory (WM). Children in grade 4 showed smaller reaction times in all conditions. However, there was no significant difference in errors between grades. Contribution of verbal and visuo-spatial WM also changed with grade. Multiplication correlated with verbal WM and performance in grade 3 but with visuo-spatial WM and performance in grade 4. We suggest that the relation to verbal WM in grade 3 indicates primary linguistic learning of and access to multiplication in grade 3 which is probably based on verbal repetition of the multiplication table heavily practiced in grades 2 and 3. However, the relation to visuo-spatial semantic WM in grade 4 suggests that there is a shift from verbal to visual and semantic learning in grade 4. This shifting may be induced because later in elementary school, multiplication problems are rather carried out via more written, i.e., visual tasks, which also involve executive functions. More generally, the current data indicates that mathematical development is not generally characterized by a steady progress in performance; rather verbal and non-verbal memory contributions of performance shift over time, probably due to different learning contents.

## Introduction

Children usually get better in arithmetic problem solving with age and experience. For instance, the processing strategy of multiplication in children changes from procedure- and strategy-based calculation to retrieval during developmental ages (Cooney et al., 1988; Lemaire and Siegler, 1995). It has been reported that there is a transition to retrieval process for solving single-digit multiplication problems in grade 4 (Cooney et al., 1988). However, this retrieval process is not constant during the following years of development (Campbell and Graham, 1985). Nonetheless, longitudinal studies for verification of this claim are scarce. In particular, the development of the automatic associations within the fact retrieval network has not been sufficiently understood.

Of major importance in multiplication verification performance is operand-relatedness. Operand-relatedness is whether the presented or responded answer belongs to the table of one of the operands or not. For instance, in a production task, an operand-related error is when a participant responds with 24 when presented with the problem 7 × 4 because 24 is part of the same multiplication table of one of the operands (here the 4). An operand-unrelated error would be the solution 30 because this number belongs neither to the multiplication table of 4 nor of 7. In a verification task for the problem 4 × 6 = 24, an operand-related verification distractor would be 4 × 6 = 28, and the operand-unrelated distractor would be 4 × 6 = 29.

It has been reported that the operand-related distractor errors make up about 87.5% of all errors in adults (Campbell, 1997; Domahs et al., 2006) and about 75.7% of all errors in children (Butterworth et al., 2003). The large frequency of operand-related errors has been explained in terms of a developing memory representation in an interrelated network of facts (Ashcraft, 1987). This representation means that during retrieval of a multiplication answer from an interconnected multiplication network, the operand-related distractors will activate the retrieval processing more than the operand-unrelated distractors and lead to a slower response with more errors. These assumptions have been implemented in the network interference model which explains that arithmetic facts are stored as nodes in an associative network in long-term memory and are retrieved via a spreading activation (Campbell, 1995). The presented multiplication generates activation in the corresponding nodes and this activation spreads along the connecting pathways to associated nodes. For example, the presentation of 7 × 3 activates node 7 along with its related nodes (14, 21, 28, etc.) and node 3 with its related nodes (6, 9, 12, etc.). In other terms, the activation of associates which are the operand-related distractors (e.g., 28 instead of 21 in the example above), increases the accessibility of these associates. Consequently, it is more plausible to verify it erroneously as a correct answer. However, in the operand-unrelated distractors (e.g., 25 instead of 21 in the example above), there is minimum activation of the associates, hereby decreasing the accessibility of them as a correct answer. Hence, activation of multiple associates interferes with the solutions because it renders these associates more accessible.

To our knowledge, there are very few longitudinal studies in regard to multiplication development in children considering operand-relatedness. For instance, in a study by Lemaire and Siegler (1995) it was shown that in three sessions of multiplication production assessment in grade 2, the proportion of both operand-related and -unrelated errors increased. The other study which used multiplication verification in children, did not report error analyses because it was stable at about 6% in grades 3 and 4 (De Brauwer and Fias, 2009). Therefore, it is still unclear if error patterns and their relation to operand-relatedness change longitudinally in children and consequently what can be inferred with regard to the longitudinal change in the multiplication fact retrieval network.

From the structure of the network interference model, two hypotheses could be brought forward for our longitudinal developmental study on multiplication facts. (i) Because the strength of the association network could increase with age and experience, the operand-relatedness error effect should be larger in older children. (ii) The alternative hypothesis would be that the network becomes more refined in reciprocal inhibition so that the single entries can be better separated with age and experience. Then, the operand-relatedness error effect should be smaller in older children. In our opinion, both views are possible. The current study set out to discern these two hypotheses.

Another main issue of this study is that to our knowledge the possible varying influence of other cognitive processes on the multiplication performance has not been studied longitudinally in children. One natural candidate for such a cognitive process is memory, containing working memory (WM) and short-term memory (STM). One account of WM capacity is defined by Shah and Miyake (1996) and Miyake and Shah (1999). In this model WM capacity contains two separate pools of domain-specific resources for verbal and visuo-spatial information. Each domain keeps and manipulates information independently from the other. This distinction between verbal and visuo-spatial domains has been supported by the previous findings (e.g., Friedman and Miyake, 2000; Miyake et al., 2001; Jarvis and Gathercole, 2003). WM has been reported as a pure measure of a child’s learning potential (Alloway and Alloway, 2010). Thus, it has been assumed to predict a child’s performance in mathematic learning based on the WM skills (Alloway and Passolunghi, 2011). While WM is defined as an ability of storage and manipulation of information, STM is considered as only storage of information for a temporary period of time (for more see Alloway et al., 2006). In other words, WM is a memory system containing separable interacting components, while STM is almost a single store (Alloway et al., 2006). In sum, STM demonstrates temporal deterioration and capacity limits, whereas WM is a multi-component system that stores and manipulates information in STM and uses attention to manage STM and applies STM to cognitive tasks (Baddeley and Hitch, 1974; Cowan, 1988; Baddeley, 1992, 1998; for more see Cowan, 2008). Therefore, STM involves a minimal load of processing, while WM contains an additional process for manipulation of information that leads to higher loading of process. Different components of STM and WM have already been reported to be involved in different mathematical tests during developing stages (see also Meyer et al., 2010) but the possibility of their different role in development of multiplication has not been longitudinally considered – therefore, the differential roles of STM and WM will also be considered in the current study.

Recent studies have shown that the relative contributions of memory components to general mathematic learning changes during development ages. At first, preschool children rely more on visuo-spatial memory than verbal memory for learning and remembering arithmetic; therefore, the best predictor of the arithmetic performance at this age is visuo-spatial sketchpad capacity (McKenzie et al., 2003; Simmons et al., 2008). Later, starting from school age, learning is more dependent on verbal rehearsal to preserve information in memory, thus recruiting more the phonological loop (Hitch et al., 1988; Rasmussen and Bisanz, 2005). This has been explained by verbally mediated strategies, in which children transform symbols and numbers into verbal code (Logie et al., 1994; Geary et al., 1996). By the first grade, performance relies equivalently on non-verbal and verbal memory. Meyer et al. (2010) showed that the verbal components of memory predict mathematical reasoning skill in grade 2, whereas the visuo-spatial component is the predictor in grade 3. Therefore, different WM and STM components seem to be critical for mathematics learning in general. However, currently we have only little data on how the different verbal and visuo-spatial components of WM and STM contribute to multiplication performance in different ages in elementary school and how the importance of such components changes over time. For our study, we hypothesized a shift between memory components, from verbal to visuo-spatial, in children during development in multiplication similarly to those reported by Meyer et al. (2010) for mathematical reasoning. In the current study as we collected longitudinal data, the first aim was to evaluate in which way children process multiplication in grades 3 and 4. According to the previous findings, we expected children in grade 4 to be faster and possibly less error-prone than in grade 3. The second aim was to investigate whether their memory processing is differentially influenced by operand-relatedness with age and experience, especially with regard to the error data. Finally, the third and main aim of this study was to investigate the contributions of verbal linguistic and visuo-spatial non-verbal representations on arithmetic skill, namely the influence of verbal and visuo-spatial STM and WM on multiplication skill.

## Materials and Methods

The current study was part of a large longitudinal project evaluating numerical development from grade 1 to grade 4. In this study, we focused on the development of multiplication performance which was measured only from grade 3 to grade 4.

### Participants

In total, 77 native German-speaking Austrian children (39 girls and 38 boys) were assessed in multiplication both at the end of grades 3 and 4. The children were between 8 years 6 months and 10 years 5 months (*M* = 9 years 4 months, SD = 7 months) in grade 3 and 1 year older in grade 4. All children had normal or corrected-to-normal vision and IQ scores in the normal range. No child received special education services or had documented brain injury or behavioral problems. This study was carried out in accordance with the recommendations of the Landesschulrat, the regional school administration, which was responsible for approval of school-related studies in Austria at that time. Parents of all subjects gave written informed consent in accordance with the Declaration of Helsinki.

### Multiplication Stimuli

Children were tested on a computerized multiplication verification task. The experiment started with eight practice trials. Multiplication problems (range of operands: 3–8; problem size: 13–54) along with the answer probe were presented at the same time on the screen in white against a black background (font: Arial; size: 48-point). Problems were presented in the form x × x = xx at the x/y coordinates (512/300) on a screen with the resolution set to 1024 × 768. In total there were 80 multiplication trials. Half of the trials were true (i.e., the solutions were displayed) and half of them were false (i.e., distractors which had to be rejected were displayed). The distractors consisted of operand-related and operand-unrelated trials. In the operand-related trials the operand split was ±1 from the solutions on the multiplication table (e.g., 6 × 3 = 21). In the operand-unrelated trials the displayed answers were not from the multiplication table. In the operand-unrelated trials the displayed answer differed from the solution by ±2 to ±9, with the average split matched at 0.4 (e.g., 6 × 3 = 13). The task was a verification paradigm where the displayed answer needed to be verified as correct or incorrect. Problem size was held approximately constant between item categories. Problems and answer probes were presented until a response was given or the response time (RT) of 15000 ms finished. The response was made by pressing the “Alt” or “Alt Gr” button of a QWERTZ keyboard to verify whether the displayed answer was the solution or distractor, respectively. It is essential to note that the solutions and distractors refer to the stimuli presented in the verification task, not the children’s responses. The children’s responses were correct or incorrect. The fixation cross was presented at the beginning of each trial for 500 ms. The inter-stimulus interval was set to 1500 ms. No feedback was given.

### Memory Tasks

Four memory components including verbal and visuo-spatial STM and verbal and visuo-spatial WM (Alloway et al., 2006; Alloway and Passolunghi, 2011) were assessed in the present study. For verbal STM, children were asked to immediately recall spoken sequences of letters (presentation rate: one letter per second). Starting with two-item sequences, sequence length was increased by one letter when at least two of three given sequences were recalled correctly; otherwise, testing was stopped. The verbal STM score was the maximum sequence length at which at least two sequences were repeated correctly. For visuo-spatial STM, in a block tapping task (Corsi, 1973), children needed to repeat pointing to cubes in the same order as the experimenter. Again, children started with two-item sequences. The procedure and scoring were identical to those in letter repetition. In general, forward span tests were defined as STM and backward span tests were defined as WM (Cowan, 1988; see also Cowan, 2008).

For verbal and visuo-spatial WM, children were asked to recall sequences of letters and blocks in reverse order. The procedure and scoring were identical to those in the STM tasks. It is noteworthy that the current study included forward recall as a measure of verbal and visuo-spatial STM and backward recall as a measure of verbal and visuo-spatial WM. In forward recall tasks the processing load is minimal as children immediately recall the sequences (Alloway et al., 2006). In contrast, in the backward recall tasks there is an additional requirement to recall the reverse sequence that imposes a substantial processing load on the child. This higher processing load has been illustrated by the finding that forward spans scores are higher than backward spans (Isaacs and Vargha-Khadem, 1989; Vandierendonck et al., 2004).

### Procedure

All children were assessed individually in one-on-one sessions in a separate room. In both grades, multiplication performance and short-term and WM were assessed.

### Analysis

Response times were measured by key-press. Only RTs for correct responses were entered into the analyses. Furthermore, response latencies shorter than 200 ms or longer than 15000 ms were not considered; however, there was no response out of this range. In a second step, responses outside the interval of ±3 SD around the individual mean were excluded. Thus, about 3% of the responses in grade 3 and about 4.5% of the responses in grade 4 were not considered for further analyses. First, we ran two repeated-measures analyses of variance (ANOVAs), first for the solution and distractor (operand-related and -unrelated together) trials for both grades and second for the operand-related and operand-unrelated distractors for both grades. Second, the correlation of the WM components was analyzed using stepwise multiple linear regression analysis on mean RTs and error rates. For the error analysis, an arcsine-square-root transformation was applied to approximate normal distribution (e.g., Winer et al., 1971).

Because of controversies regarding confirmation of null hypothesis using traditional statistical inference, the Bayesian method was used in the current study. The method described in detail by Masson (2011) enables calculating graded evidence for null hypothesis (i.e., no difference between groups) and alternative hypothesis (i.e., difference between groups). In the analysis, sum of squares and number of observations from ordinal ANOVA were used to calculate Bayesian factors which then can be used to calculate posterior probabilities (see also Raftery, 1995). In fact, we employed the Bayesian method in order to estimate the likelihood of correctness of the null and alternative hypotheses.

## Results

Trials with RTs 3 SDs above or below a child’s average RT were excluded. Children with trial exclusion or an error rate of more than 33% were not considered [six children (mean age = 9 years 4 months, two girls and four boys)]. Thus, the data of 71 children was considered in the analyses. Children had on average significantly higher WM scores in grade 4 than in grade 3 (see **Table 1**). A previous study suggested that the window between second and third grades is too short a time frame for major changes in WM capacity (Meyer et al., 2010) but interestingly we found that this difference is statistically significant between grades 3 and 4.

**Table 1:** Means and SDs of memory components.

	Grade 3		Grade 4			
Variable	*M*	SD	*M*	SD	*t*^a^	*p*^b^
Verbal short-term memory (STM)	4.55	0.73	4.92	0.73	-4.68	<0.001
Verbal working memory (WM)	2.89	0.60	3.30	0.55	-4.72	<0.001
Visuo-spatial STM	5.06	0.70	5.56	0.67	-4.88	<0.001
Visuo-spatial WM	4.18	1.10	4.69	0.86	-3.82	<0.001

*^a^Paired sample t test. ^b^Two-tailed significance level of 0.01.*

### Solution vs. Distractor

First, we investigated the effect of grade on the solution and distractor (both operand-related and -unrelated together) trials for RTs and accuracy.

#### Response Times

**FIGURE 1:**
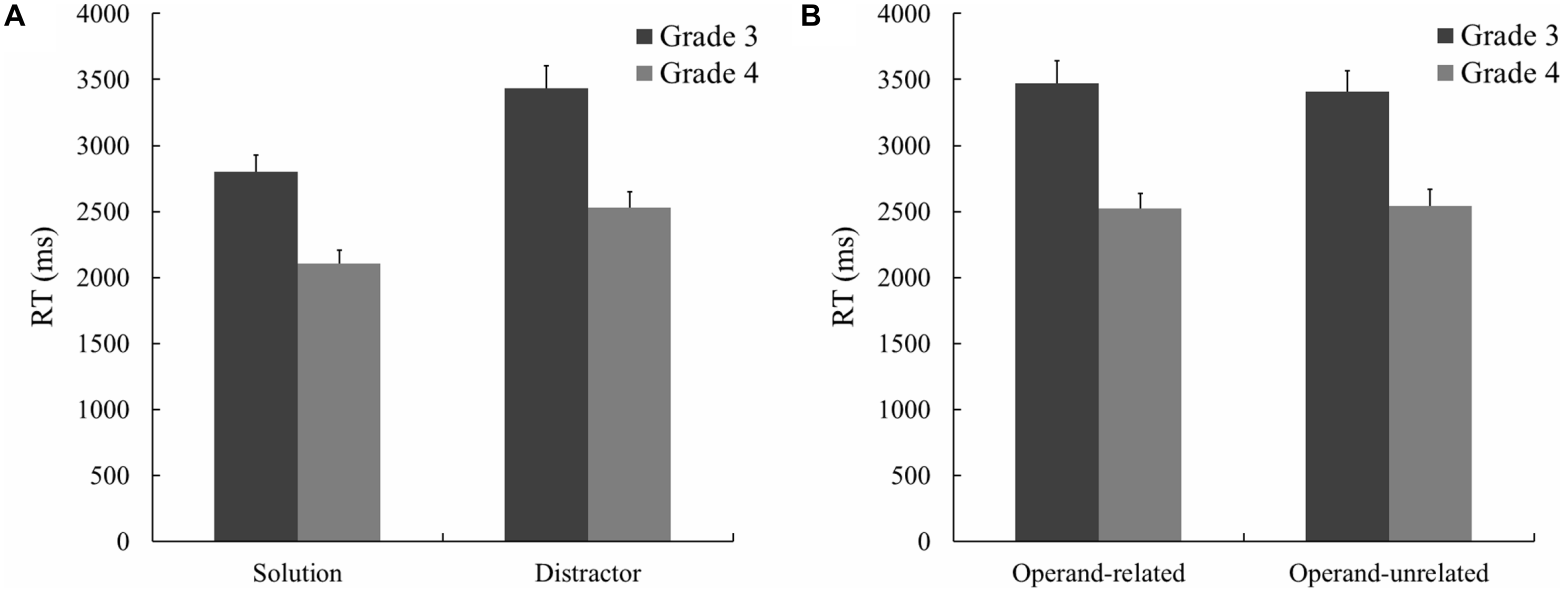
**(A)** Mean response times (RTs; in ms) for the solution and distractor. **(B)** Mean RTs (in ms) for the operand-related and -unrelated distractors. Error bars reflect SEs.

Raw RT of correct responses was analyzed by repeated-measures ANOVA with grade (3 or 4) and condition (solution or distractor) as within-participant factors. Children took on average 3118 ms (SD = 1243 ms) to choose the correct answer in grade 3 and 2320 ms (SD = 916 ms) in grade 4. Children in grade 4 were on average 798 ms faster than in grade 3, *F*(1,70) = 58.46, *p* < 0.001, $\eta_{p}^{2}$ = 0.46. RTs for the solution condition was 531 ms faster than for the distractor condition which indicated a significant difference between the two conditions, *F*(1,70) = 162.07, *p* < 0.001, $\eta_{p}^{2}$ = 0.70. Interaction of grade × condition showed that the effect of grade is greater for the distractor than for the solution, *F*(1,70) = 9.14, *p* = 0.003, $\eta_{p}^{2}$ = 0.12 (**Figure 1A**; **Table 2**). Bayesian analysis revealed that the posterior probability of null hypothesis for grade and condition was about zero (the same probability of alternative hypothesis was complementary, i.e., about 1). The posterior probability of null hypothesis for interaction was 0.10 (the same probability of alternative hypothesis was 0.90).

**Table 2:** Mean response times (RTs) and error rates (and SDs) for multiplication trials.

		Grade 3		Grade 4	
		*M*	SD	*M*	SD
RT (ms)	Solution	2799	1091	2108	847
	Operand-related distractor	3468	1466	2523	948
	Operand-unrelated distractor	3406	1371	2544	1045
Errors (%)	Solution	6.30	6.24	5.77	6.04
	Operand-related distractor	7.68	9.41	8.94	10.52
	Operand-unrelated distractor	4.15	7.37	5.56	8.17

#### Error Rates

Error rates were analyzed by repeated-measures ANOVAs with grade (3 or 4) and condition (solution or distractor) as within-participant factors. Overall, children responded incorrectly on 6.11% of all trials in grade 3 and on 6.51% in grade 4. Error rates did not differ significantly neither between the grades, *F*(1,70) = 0.11, *p* = 0.74, $\eta_{p}^{2}$ = 0.002, between the conditions, *F*(1,70) = 0.095, *p* = 0.76, $\eta_{p}^{2}$ = 0.001, nor in their interaction, *F*(1,70) = 3.04, *p* = 0.09, $\eta_{p}^{2}$ = 0.042. Thus, the RT differences could not be explained by speed-accuracy trade-offs. Bayesian analysis revealed that the posterior probability of null hypothesis for grade and condition was 0.89 (the same probability of alternative hypothesis was 0.11). The posterior probability of null hypothesis for interaction was 0.65 (the same probability of alternative hypothesis was 0.35). This is rated as positive evidence for the null hypothesis applying the criteria suggested by Masson (2011).

### Operand-Related vs. Operand-Unrelated

Second, we investigated the effect of grade on the operand-related and operand-unrelated distractor trials for RTs and accuracy. Note that this analysis was done for the distractors only.

#### Response Times

Raw RT of correct responses was analyzed by repeated-measures ANOVA with grade (3 or 4) and condition (operand-related or operand-unrelated) as within-participant factors. Children in grade 4 were on average 903 ms faster than in grade 3, *F*(1,70) = 53.74, *p* < 0.001, $\eta_{p}^{2}$ = 0.43. Raw RT neither differed significantly between conditions, *F*(1,70) = 0.28, *p* = 0.60, $\eta_{p}^{2}$ = 0.004, nor did interaction between conditions and grade, *F*(1,70) = 1.57, *p* = 0.22, $\eta_{p}^{2}$ = 0.022, (**Table 2**; **Figure 1B**). Bayesian analysis revealed that the posterior probability of null hypothesis for grade was about zero (the same probability of alternative hypothesis was about 1). However, the posterior probability of null hypothesis for condition was 0.88 (the same probability of alternative hypothesis was 0.12); and for interaction 0.79 (the same probability of alternative hypothesis was 0.21).

#### Error Rates

Error rates were analyzed by repeated-measures ANOVAs with grade (3 or 4) and condition (operand-related or operand-unrelated) as within-participant factors. The operand-related distractor trials were significantly more error-prone than the operand-unrelated distractor, *F*(1,70) = 22.82, *p* < 0.001, $\eta_{p}^{2}$ = 0.25. Error rates neither differed significantly between the grades, *F*(1,70) = 1.43, *p* = 0.24, $\eta_{p}^{2}$ = 0.02, nor did interaction between conditions and grade, *F*(1,70) = 0.06, *p* = 0.81, $\eta_{p}^{2}$ = 0.001. Bayesian analysis revealed that the posterior probability of null hypothesis for grade was 0.80 (the same probability of alternative hypothesis was 0.20). However, the posterior probability of null hypothesis for condition was about zero (the same probability of alternative hypothesis was about 1); and for interaction 0.89 (the same probability of alternative hypothesis was 0.11).

### Relation between Multiplication Performance and Memory Components

#### Regression Analysis^1^

In order to investigate which memory component predicted multiplication performance in grades 3 and 4, a series of stepwise regression analyses were conducted. For each grade, one regression predicted each of the 10 verification dependent variables (total RT, solution RT, distractor RT, operand-related distractor RT, operand-unrelated distractor RT, total error, solution error, distractor error, operand-related distractor error, and operand-unrelated distractor error) from the four memory components measured concurrently. All four memory scores were entered simultaneously with a stepwise function. This approach allowed us to identify the best predictors for different dependent variables in both grades. The model of total errors in grade 3 comprised only the predictor verbal WM, *R*^2^ = 0.057, adjusted *R*^2^ = 0.044, *F*(1,69) = 4.193, *p* = 0.044, while the other memory components failed to explain significant amounts of additional variance. Inspection of the individual beta weights indicated a significant influence of verbal WM (**Table 3**). The model of the operand-unrelated distractor errors in grade 3 comprised only the predictors verbal WM and verbal STM, *R*^2^ = 0.178, adjusted *R*^2^ = 0.153, *F*(2,68) = 7.340, *p* = 0.001, while the other memory components failed to explain significant amounts of additional variance. Inspection of the individual beta weights indicated a significant influence of verbal WM and verbal STM (**Table 3**). The model of total errors in grade 4 comprised only the predictor visuo-spatial WM, *R*^2^ = 0.072, adjusted *R*^2^ = 0.058, *F*(1,69) = 5.325, *p* = 0.024, while the other memory components failed to explain significant amounts of additional variance. Inspection of the individual beta weights indicated a significant influence of visuo-spatial WM (**Table 3**). All other predictors and criterion variables were not significant in regression analyses. Bayesian analysis revealed that the posterior probability of null hypothesis for total error in grade 3 was 0.51 (the same probability of alternative hypothesis was 0.49). However, the posterior probability of null hypothesis for the operand-related distractor error was about zero (the same probability of alternative hypothesis was about 1); and for total error in grade 4 0.38 (the same probability of alternative hypothesis was 0.62).

**Table 3:** Results for significant predictors entered in the stepwise multiple regression analysis.

Grade	Variable	Predictor	*B*	Standardized β	*t*	*p*
3	Total error	Verbal WM	-0.049	-0.239	-2.048	0.044
	Operand-unrelated	Verbal STM	0.095	0.408	3.603	0.001
	distractor error	Verbal WM	-0.069	-0.242	-2.132	0.037
4	Total error	Visuo-spatial WM	-0.041	-0.268	-2.308	0.024

## Discussion

In the current study we collected longitudinal data from children in grades 3 and 4. The first aim of the study was to evaluate how children process multiplication in different grades. The second aim was to investigate the development of the multiplication fact retrieval network, i.e., whether their memory of multiplication facts is influenced by operand-relatedness. Furthermore, the third and main aim of this study was to investigate the contributions of verbal and visuo-spatial STM and WM to multiplication skill.

### Multiplication Fact Fluency Increases Longitudinally with Age and Experience

As we expected, children in grade 4 were faster than in grade 3 which is in line with previous findings that children become faster during development (Koshmider and Ashcraft, 1991; Lemaire et al., 1996; Butterworth et al., 2003; De Brauwer and Fias, 2009). Although children in both grades depended heavily on memory retrieval to solve the simple one-digit problems, this retrieval processing was more dominant in grade 4 (Verguts and Fias, 2005). Thus, because of the faster processing, verification of the solution, and rejection of the distractor was faster.

As regards RTs, children in both grades verified the solutions faster than the distractors (Koshmider and Ashcraft, 1991; De Brauwer and Fias, 2009). Koshmider and Ashcraft (1991) explained this result by saying that the solutions facilitate verification of the correct answer in children when the solutions are used as a prime, probably because the solutions make the strongest activation in the related nodes which in turn accelerates memory retrieval process.

As regards errors, the difference of error rate between the solutions and distractors was not statistically significant in the current study: the error rates remained stable, about 6% in grades 3 and 4. Again, this non-significant change in error rates is in line with previous results (Koshmider and Ashcraft, 1991; De Brauwer and Fias, 2009).

In brief, children in grade 4 were faster in both conditions than in grade 3 but their performance in regard to error did not differ significantly. This can be explained by more efficient and faster solving strategies with age which are, however, not yet more accurate than the slower strategies of younger children.

### No Changes in the Operand-Relatedness Effect with Age and Experience

In line with our main hypothesis, the operand-related distractors were erroneously responded to significantly more frequently than the operand-unrelated distractors. The finding is in line with the previous studies in children (Koshmider and Ashcraft, 1991; Lemaire and Siegler, 1995; Butterworth et al., 2003) which reported operand-related errors as the most frequent errors. It implies that multiplication facts are stored in the associative network already 1 year after the first multiplication facts are learnt. The suggestion of the interacting neighbors model even holds for those young children in grades 3 and 4. The model assumes that the operand-related distractors lead to stronger confounding with the solutions than the operand-unrelated distractors.

However, as regards the operand-relatedness effect, we found no difference between grades 3 and 4. In fact, there was an operand-relatedness effect in both grades but it was neither stronger nor weaker than in the other grade. This result was again in line with the only longitudinal study of multiplication in a verification paradigm in children (De Brauwer and Fias, 2009). The finding of the present study is consistent with the idea that multiple changes may occur in the associative network. First, the strength of the association network increases with age and experience (which leads to faster retrieval in older children). Second, the network may become more refined in reciprocal inhibition. More association strength with age would lead to a higher operand relatedness effect because related entries are activated more. However, better reciprocal inhibition would lead to better differentiation between entries and therefore to a lower operand relatedness effect because related entries could be more easily inhibited. If both processes increase similarly with age and experience, the operand-relatedness effect may stay unchanged. This is what we found in the present study.

### An Age-Related Shift from Verbal to Visuo-Spatial Working Memory Predicting Multiplication Performance

Interestingly, we found that verbal WM predicts multiplication problem solving in grade 3, while in grade 4 visuo-spatial WM is the predictor. This finding for multiplication performance extends and refines current accounts of the role of different WM components during different developing stages. A developmental change of the influence of verbal and visuo-spatial components was reported several years ago for more general math capabilities: it was shown that there is a strong link between verbal and mathematical skills when young children are learning new information which becomes weaker in older children as the result of practice (Jensen, 1980). In accordance to this finding, several studies have shown the weak conjunction between phonological loop and mathematical performance in adults (e.g., Logie and Baddeley, 1987; Heathcote, 1994; Logie et al., 1994). The present study did not find any significant correlation between verbal WM and multiplication performance in grade 4 which can be related to a gradual shift from strongly verbal representations of multiplication to the build-up of a more abstract semantic retrieval of mathematical facts from long-term memory which is visually based, at least when the stimuli are presented visually as in our study.

One possible suggestion is that one may expect to see more predictability of verbal WM in grade 4. However, this was not the case. Three reasons may explain this finding. First, learning and task context of multiplication problems encountered in (Austrian) schools may contribute to their explanation. While in the initial learning phase in grades 2 and 3, multiplication problems may be more auditorily and verbally trained, they may be more often encountered visually as part of more complex arithmetic problems in grade 4. Second, the shift toward more visuo-spatial processing is consistent with previous studies on arithmetic development showing that in children, arithmetic tasks require superior demand of visuo-spatial processing during the development (Alloway and Passolunghi, 2011). In fact in adults, Fürst and Hitch (2000) showed that the phonological loop is not crucially caught up in retrieving factual mathematical knowledge which is also consistent with our data that verbal WM plays a lesser role in older children. Finally, the same verbal to visuo-spatial WM shift has been observed in other arithmetic domains. Meyer et al. (2010) found such a shift from grade 2 to grade 3 in some basic arithmetic and mathematical reasoning. For these reasons, we believe that our finding of a developmental shift from verbal to visuo-spatial WM with age and experience does not come as a surprise but is actually consistent with the literature in other fields of arithmetic development. In sum, the data shows an important developmental shift from verbal to visuo-spatial WM in the prediction of simple multiplication problem performance (as indexed by overall errors) from grade 3 to grade 4.

Furthermore, neuroimaging studies revealed a neural dissociation of verbal and visuo-spatial WM (Smith et al., 1996; Thürling et al., 2012), which were modified differently due to arithmetic training. The brain activation pattern of development and training of calculation shows a shift of activation from the frontal to the parietal regions (for a review Zamarian et al., 2009). This modification shows a shift from verbally representation of the calculation to more visually representation. While the frontal are is involved in verbal WM, the parietal area is mostly involved in visuo-spatial WM (for a review Cabeza and Nyberg, 2000; Dumontheil and Klingberg, 2012).

Interestingly, for the operand-unrelated distractor errors in grade 3, verbal STM reached significance as the only STM predictor in our whole study. However, this makes sense because during the second and third years of elementary school children are commonly highly trained with direct verbal learning of multiplication facts. Therefore, verbal STM is still significant for multiplication in grade 3. In the fourth grade, however, children have to use the learnt skills, such as multiplication, indirectly in more advanced mathematic problems such as mathematical text questions which does not involve any aspect of STM massively in this grade. Verbal STM may only affect the operand-unrelated distractor errors because the operand-relatedness may lead to interference specifically in the STM where no information is manipulated. Vice versa, the solutions share at least one element with possible operand-related distractors. It seems plausible that in such clear cases which require no manipulation and selection of information, verbal STM processing is most predictive. Again, our finding that verbal STM influences multiplication performance in earlier grades is consistent with previous findings from other more general arithmetic measures. For instance, Alloway and Passolunghi (2011) showed that verbal and visuo-spatial STM were involved in arithmetic performance at age 7 but only visuo-spatial STM was involved at age 8. Although the prediction of operand-unrelated distractor error by verbal STM in grade 3 was reasonable, the positive correlation between verbal STM and operand-unrelated distractor error was unexpected. One possible explanation would the interference of other simultaneous processes, which occupy STM. We know that the results of simple multiplication problems are retrieved from long-term memory (for a review of neuroimaging studies see Zamarian et al., 2009). Indeed, the results of the one-digit time one-digit multiplication problems, which belong to the multiplication table are stored in long-term memory and retrieved via WM. Therefore, it may conclude that to answer these problems, we do not rely so much on STM (Butterworth et al., 1996). Hence, any involvement of STM in other simultaneous processes can interfere with this fact retrieval procedure. But this is not the case of WM. We know that WM is involved in almost every cognitive process. Since WM has a crucial role in the retrieving of multiplication result, higher WM capacity can lead to a better manipulation on different processing including multiplication performance. Butterworth et al. (1996) showed that in a patient with impaired STM, the mental calculations such as one-digit multiplication are intact. However, we believe that this is only a possible interpretation, which needs to be tested directly.

None of the memory components were able to predict RTs in both grades. We believe that this is due to high (inter-individual and intra-individual) variability in the RT measures for the children, which may be overcome in comparisons of means but may be critical for inter-individual comparisons and correlations. Variability in RTs can be explained by several sources. First, children use different strategies for multiplication problem solving (Cooney et al., 1988; Sherin and Fuson, 2005) which mostly lead to equal (correct) responses but to different RTs. Second, individual differences in mathematical competence modulate RTs during mental arithmetic. For instance, Grabner et al. (2007) suggested that the recruitment of retrieval strategies during arithmetic problem solving may be caused by individual differences in mathematical ability. Therefore, different children rely on different memory processes. This may lead to highly variable RTs, not only intra-individually, but also inter-individually, even though both ways may lead to the solution of the multiplication problem. For these reasons, RT may be more sensitive to intra- and inter-individual variability than errors. Future studies should probably combine investigations of the strategy used and different WM components to examine if specific WM components are associated with specific solving strategies.

## Conclusion

In line with the previous findings (Swanson, 2006; Meyer et al., 2010), the current study suggests that although verbal WM may facilitate early stages of arithmetic learning and performance, visuo-spatial WM may support later arithmetic performance during the development – at least during elementary school. We would like to mention that while we found this shift in prediction of multiplication problem solving from grade 3 to 4, the others found it in different ages, however, albeit for different mathematical contents. For instance Meyer et al. (2010) found the shift in mathematical reasoning from grade 2 to 3. Meyer et al. (2010) were concerned with mathematical reasoning. Their mathematical reasoning subtest of the WIAT-II “is a verbal problem solving test that measures the ability to count, identify geometric shapes and solve single- and multi-step word problems.” In contrast, we were concerned with multiplication. Multiplication – as said above – is introduced in grade 2, verbally trained in grade 3 and then integrated in visual tasks in grade 4 – therefore the shift from verbal to visual makes sense for multiplication at exactly that age. Because the mathematical reasoning subtest of the WIAT-II is an aggregate score of many different tasks, it is hard to tell, why the shift was caused in Meyer et al. (2010) from grade 2 to 3. However, because the subtests contained some very basic tasks like counting or identifying geometric shapes, which are introduced earlier than multiplication, it is possible that the shift from verbal to visuo-spatial WM is also earlier in their study. In sum, it seems that this shift may be found in different developing ages for differing mathematical skills. This shift may serve as an essential step in mathematical development, however, its relation to age may vary according to mathematical content – in our view, this deserves further more detailed investigation in the future.

This changing role of verbal and visuo-spatial WM components for predicting arithmetic performance could be useful for diagnosis and intervention in children with mathematical learning difficulties. However, we recommend that future studies should also assess children’s strategy-use. By examining strategy-use together with the contribution of different memory components, researchers might be able to uncover cognitive demands of multiplication learning in developmental ages.

As regards the fact retrieval network itself, the current data suggest that retrieval is faster and more efficient from grade 3 to grade 4; however, the lack of change in the operand-relatedness effect with age may suggest that in children’s fact retrieval network both automatic association and reciprocal inhibition of concurrent responses may increase. More associations and at the same time better inhibition might lead to an unaltered operand-relatedness effect in this longitudinal study. This is only a speculative interpretation which needs to be examined in future studies with considering inhibitory control, attentional processing, and self-regulation as well.

## Conflict of Interest Statement

Theauthors declare that the research was conducted in the absence of any commercial or financial relationships that could be construed as a potential conflict of interest.

## Footnotes

1 We know from many previous numerical and arithmetic experiments that RT data in children are very noisy. Hence, employing z-transformed RT to reduce inter-individual differences in intra-individual variance (cf. Nuerk et al., 2004, and many following papers since), we reanalyzed linear regressions. In general, none of the memory components predicted z-transformed RTs in grade 3. In grade 4 the verbalWMcomponent predicted solution z-transformed RT, distractor z-transformed RT, and operand-related distractor z-transformed RT. However, this suggests that intra-individual noise in the RT data may at least partially account for the null effects observed in RTs.
